# 

**Published:** 1899-10-21

**Authors:** 


### NOTICE TO CORRESPONDENTS.

All MS., Letters, Books for Review, and other matters intended for the Editor should be addressed The Editor, "The Hospital," The Hospital Building, 28 & 29, Southampton Street, Strand, W.C.

The Editor cannot undertake to return rejected MS., even when accompanied by stamped directed envelope.

**Notice to Advertisers and Subscribers.**

All Advertisements, Orders for Copies of The Hospital, and Business Communications should be addressed to **THE MANAGER (Not to the Editor),** The Hospital, The Hospital Building, 28 & 29, Southampton Street, Strand, London, W.C.

The Cost of Subscription, post free, is as follows:—Per week, 2½d.; per quarter, 2s. 8d.; per half-year, 5s. 3d.; per annum, 10s. 6d. Foreign:—Per annum, 15s. 2d., payable, in all cases, in advance.

**NOTICE.—Vol. XXVI. of "The Hospital" (April, 1899, to September, 1899), in handsome cloth binding, will shortly be on sale at the Office of this Journal, price 7s. 6d. Cases for binding the Numbers contained in this Volume 1s. 6d. Index to Vol. XXVI., 2½d., post free.**

**The Monthly Part for October is now ready. Price 6d., by post 8d.**

### NOTES ON BOOKS.

At the time of their delivery we noticed the Harben Lectures on "The Administrative Control of Tuberculosis," by Sir Richard Thorne Thorne, K.C.B. Since then they have been published in book form (Baillière, Tindall, and Cox), and we have no doubt that they will be much consulted, showing as they do what may properly be expected from State interference in the matter, and suggesting, as they must do to the reader who bears in mind the high position occupied by the author in the official world, certain very definite lines as being the limit beyond which such State interference is not likely to be carried, at any rate for the present.

The first number of Climate (Livingstone College, Stratfort, E.), a new quarterly edited by Dr. Harford Battersby, contains various interesting articles chiefly dealing with travel in various parts of the world—Africa, Japan, and Labrador. It is nicely printed and illustrated, and as it develops so as to cover a larger field—and what field is too wide to be covered by the title Climate?—it ought to be a success.

In these warlike days even the Art Portfolio devotes its illustrations to matters redolent of fighting, the Battle of Trafalgar, the Death of Nelson, and an admirable portrait of Nelson being among its illustrations.

### BOOKS RECEIVED.

The Ideal Publishing Union.

"Experimental Proofs of the Role of Alcohol." By E. MacDowel Cosgrave, M.D.

M. Harland and Son.
"Annual Report." By J. Wright Mason, M.B., D.P.H., Medical Officer of Health, Kingston-upon-Hull.

John Wright and Co.
"A Guide to Urine Testing." By Mark Robinson, L.R.C.P., L.R.C.S.

Henry J. Glaisher.
"Asthma: Recent Developments in its Treatment." By Ernest Kingscote, M.B., C.M.

Church Lads' Brigade.
"Manual for the Church Lads' Brigade Medical Corps."

**Periodicals and Pamphlets.**—Sunday at Home—Leisure Hour—Boy's Own Paper—Charity Organisation Review—Sanitary Record—Literary Digest—Medical Record—Medical Press—Asylum News—Guy's Hospital Gazette—St. Bartholomew's Hospital Journal—New York Medical Record—Hospital Saturday Fund Journal—Vectis—Health—London Hospital Gazette—Zenana.

### SCRAPS AND GLEANINGS.

Last year 2,483 cases were treated at the Sunderland Infirmary and 241 operations were performed.

The Hospital Saturday Committee collected £90 during the past year for the Mirfield Memorial Hospital.

The Lincoln County Hospital has benefited by £686 from the Hospital Saturday Committee's contribution this year.

This year has produced the record Hospital Saturday for the Memorial Cottage Hospital at Llandudno, the sum collected being £133 11s. 3d.

The sum realised this year as a result of the street collections on behalf of the Rotherhithe Cottage Hospital was £42 17s. 2d., which is the record street collection in Rotherhithe.

The total cost of the isolation hospital for Dundee is estimated at £6,500 or £7,000. The buildings consist of an administrative block, the isolation blocks, and a discharge block.

The number of extern and intern patients this year at the National Maternity Hospital, Dublin, was 4,966, having increased to this number from 2,516 in the first year of its establishment.

It was unanimously resolved at a recent meeting of the Gladesville Progress Association to aid in bringing to a successful issue the movement to establish a cottage hospital for the Ryde electorate.

The Birmingham Hospital Sunday collection this year is to be for the Queen's Hospital, the expenditure of which now amounts to £10,000, while the annual income from subscriptions is £2,800 and from endowments £871.

In aid of the British Ophthalmic Hospital at Jerusalem, which is at present specially in need of assistance, an amateur theatrical performance will be held at the Royal Lyceum Theatre on the afternoon of December 2nd.

Last year's collections of the Birmingham Hospital Saturday Fund amounted to £17,910, £10,000 of which has been distributed to the medical charities of the city as a free gift on account of the relief and maintenance of patients.

This year's flower stall demonstration on behalf of the St. John's Hospital and the Richmond Hospital was worked up by the kindly helpers with as much enthusiasm as ever, and appears to have been very successful. At the two stalls upwards of £21 was taken on the Saturday of the demonstration.

The number of fever patients from Chester and the neighbouring counties received into the Chester Infirmary has averaged during the last ten years 160 per year. The fever patients are now to be all transferred to the fever hospital, and with this transference a certain proportion of the funds will also be taken away from the infirmary.

To assist in raising the £12,000 required for the building and furnishing of four additional residential homes for patients in connection with the National Hospital for Consumption for Ireland, an open-air fête was recently held in Mr. Walter Tighe's grounds at Milltown, near Rathnew, which were kindly lent by the owner for the occasion. A great many attractions were offered, including theatricals, a concert, phonograph expositions, art gallery, &c.

The following changes have been made in the rules of the Rotherhithe Cottage Hospital: (1) New medical patients (women and children only) will be required to purchase a letter (price 6d.), which will entitle the holder to three months' medical attendance. (2) Surgical patients will be required to pay one penny each attendance. Those who are unable to pay as above should apply to the Sister-in-charge, who has a certain number of free letters in hand, as also have subscribers.

### The Student of Medicine.

### APPOINTMENTS.

E. Buxton, M.D., F.R.C.S.E., M.R.C.P.E., D.P.H., appointed Medical Officer of Health to the Little Crosby and Hightown Districts. H. Faulkner, M.B., Ch.B.Edin., appointed Assistant House Physician to the General Hospital, Birmingham. W. J. Forbes, M.B., B.Ch., B.A.O., R.U.I., appointed Medical Officer of Health for the Knaresborough Rural District. W. McLean, M.R.C.S., appointed Surgeon R.M.S. "Dunottar Castle," Castle Mail Packets Company. A. M. Mitchell, M.A., M.D., B.C.Cantab., D.P.H.Camb., appointed Honorary Assistant Medical Officer to the Royal Surrey County Hospital, Guildford. F. C. Moore, M.D., M.Sc.Vict., appointed Pathological Registrar to the Royal Infirmary, Manchester. S. Southall, M.B., Ch.B.Edin., appointed House Surgeon to the General Hospital, Birmingham. H. Thorp, M.R.C.S.Eng., L.R.C.P.Lond., appointed Junior House Surgeon to the Warrington Infirmary and Dispensary. J. A. Ward, M.R.C.S.Eng., L.R.C.P.Lond., appointed Medical Officer to the Grays Urban District Council. A. B. Webb, M.B., Ch.B.Melb., appointed Health Officer for the Shire of Ballan, Victoria.

### NOW READY,

A REPRINT OF

### A NURSING HANDBOOK

TOGETHER WITH S. MAW, SON, AND THOMPSON'S

### COMPLETE CATALOGUE OF NURSING REQUISITES.

The above work is now republished at a cost of 1s. per copy. Messrs. S. M., S., and T. have been compelled to make this charge on account of the extreme popularity of the work and the great expense incurred in its production.

***Post Free on receipt of 1s.***

### S. MAW, SON, and THOMPSON,

7 to 12, ALDERSGATE STREET, LONDON, E.C.

